# Ring-Substituted 1-Hydroxynaphthalene-2-Carboxanilides Inhibit Proliferation and Trigger Mitochondria-Mediated Apoptosis

**DOI:** 10.3390/ijms21103416

**Published:** 2020-05-12

**Authors:** Tereza Kauerová, Tomáš Goněc, Josef Jampílek, Susanne Hafner, Ann-Kathrin Gaiser, Tatiana Syrovets, Radek Fedr, Karel Souček, Peter Kollar

**Affiliations:** 1 Department of Pharmacology and Toxicology, Faculty of Pharmacy, Masaryk University, Palackeho tr. 1946/1, 612 42 Brno, Czech Republic; tereza.kauerova@gmail.com; 2 Department of Chemical Drugs, Faculty of Pharmacy, Masaryk University, Palackeho tr. 1946/1, 612 42 Brno, Czech Republic; t.gonec@seznam.cz; 3 Institute of Neuroimmunology, Slovak Academy of Sciences, Dubravska cesta 9, 845 10 Bratislava, Slovakia; josef.jampilek@gmail.com; 4 Institute of Pharmacology of Natural Products and Clinical Pharmacology, Ulm University, Helmholtzstraße 20, 89081 Ulm, Germany; susanne.hafner@uni-ulm.de (S.H.); ann-kathrin.fuchs@uni-ulm.de (A.-K.G.); tatiana.syrovets@uni-ulm.de (T.S.); 5 Department of Cytokinetics, Institute of Biophysics of the Czech Academy of Sciences, Kralovopolska 135, 612 65 Brno, Czech Republic; fedr@ibp.cz (R.F.); ksoucek@ibp.cz (K.S.); 6 Department of Human Pharmacology and Toxicology, Faculty of Pharmacy, University of Veterinary and Pharmaceutical Sciences Brno, Palackeho tr. 1946/1, 612 42 Brno, Czech Republic

**Keywords:** salicylanilides, hydroxynaphthalene carboxamides, apoptosis, cell cycle, antiproliferative effect

## Abstract

Ring-substituted 1-hydroxynaphthalene-2-carboxanilides were previously investigated for their antimycobacterial properties. In our study, we have shown their antiproliferative and cell death-inducing effects in cancer cell lines. Cell proliferation and viability were assessed by WST-1 assay and a dye exclusion test, respectively. Cell cycle distribution, phosphatidylserine externalization, levels of reactive oxygen or nitrogen species (RONS), mitochondrial membrane depolarization, and release of cytochrome c were estimated by flow cytometry. Levels of regulatory proteins were determined by Western blotting. Our data suggest that the ability to inhibit the proliferation of THP-1 or MCF-7 cells might be referred to *meta*- or *para*-substituted derivatives with electron-withdrawing groups -F, -Br, or -CF_3_ at anilide moiety. This effect was accompanied by accumulation of cells in G1 phase. Compound **10** also induced apoptosis in THP-1 cells in association with a loss of mitochondrial membrane potential and production of mitochondrial superoxide. Our study provides a new insight into the action of salicylanilide derivatives, hydroxynaphthalene carboxamides, in cancer cells. Thus, their structure merits further investigation as a model moiety of new small-molecule compounds with potential anticancer properties.

## 1. Introduction

The class of salicylanilide derivatives has been attracting considerable interest for many years, but nowadays renewed research in the field of salicylanilides pharmacological properties has revealed their novel biological effects [1]. Over the past few years, hydroxynaphthalene carboxamides as a group of salicylanilide derivatives have been studied for their promising antimycobacterial properties [2,3,4,5,6]. Some ring-substituted 1-hydroxynaphthalene-2-carboxanilide derivatives have been previously reported to exert the antimycobacterial activity in vitro against three strains, namely *Mycobacterium marinum*, *Mycobacterium kasasii*, and *Mycobacterium smegmatis*, that are model pathogens for *Mycobacterium tuberculosis* [2]. However, the biological effects of salicylanilides are not limited to antimycobacterial effects. Their derivatives also possess a large spectrum of other various anti-infective activities such as antibacterial, anthelmintic or antifungal [7,8,9,10,11,12,13,14] and subsequent research of their new pharmacological properties also pointed out the potential antitumor activity of salicylanilide derivatives among which niclosamide could be mentioned. This broad-spectrum anthelmintic agent is receiving new attention when it is currently being studied for repurposing in oncology [15,16]. Until now, its anticancer potency has been thoroughly investigated and a large number of studies have established such effects using both in vitro and in vivo models [17,18,19,20]. The efforts to elucidate the mechanism of action represent a key feature of new anticancer drug discovery. In general, salicylanilides were primarily found to impair mitochondrial function as uncouplers of oxidative phosphorylation [21]. In the field of their potential anticancer properties, a few recent studies showed that the activity of niclosamide against cancer cells can be partly mediated through targeting mitochondria with its membrane depolarization or generation of reactive oxygen species (ROS) [22,23,24,25].

Nevertheless, the anticancer activity of salicylanilide derivatives is most likely related to multiple mechanisms. Until now, salicylanilides were found to inhibit EGFR tyrosine kinase activity due to the ability of their structure to form an intramolecular hydrogen bond, and thus mimic the pyrimidine ring in the structure of quinazoline EGFR inhibitors such as gefitinib [26,27]. Another studies described promising inhibitors of poly(ADP-ribose) glycohydrolase (PARG) containing modified salicylanilide structure [28], or salicylanilide inhibitor of erbB-2 tyrosine kinase phosphorylation [29]. Evidence from several reports has indicated that besides targeting mitochondria, niclosamide regulates multiple cellular pathways such as Wnt/β-catenin, NF-κB, or mTORC1 that are involved in the initiation, progression, or metastasis of cancer [19,23,30,31,32,33]. Moreover, niclosamide was identified as a new small-molecule inhibitor of the STAT3 signaling pathway, and thus became a lead molecule with salicylanilide scaffold for the development of inhibitors of STAT3 signaling pathway [34,35].

Taking into consideration the pharmacological potential of salicylanilides, the structure of hydroxynaphthalene carboxamides as their derivatives was recently designed. Their molecule was formulated by the extension of salicylanilide structure with the additional aromatic ring based on bioisosterism with quinoline like compounds [2,6,9]. That structure is also considered to be a privileged scaffold in cancer drug discovery [36]. Our previous studies on monocytic leukemia THP-1 cells showed cytotoxic effects of hydroxynaphthalene carboxamides substituted by electron-withdrawing groups on anilide ring [2,6,9]. In our follow-up study, we have reported the antiproliferative activity of nitro-substituted hydroxynaphthalene carboxamides in two different cancer cell lines THP-1 and MCF-7 that was associated with the modulation of cell cycle progression and induced apoptosis in THP-1 cells [37]. Additionally, the structure of hydroxynaphthalene carboxamides was recently identified as a new model moiety for the development of BRAF kinase inhibitors [38]. In the present study, we aimed to investigate the effects of the group of halogenated hydroxynaphthalene carboxamides **1**–**10** (Table 1) on proliferation and cell cycle distribution of THP-1 and breast cancer MCF-7 cells. In addition, we evaluated their cytotoxic effects in terms of induced mitochondria-mediated apoptosis.

## 2. Results

### 2.1. Proliferation Inhibitory Effects and Cytotoxicity Induced by 1-Hydroxynaphthalene-2-Carboxanilides in THP-1 and MCF-7 Cells

Initially, the proliferation and viability of two different cell lines, THP-1 and MCF-7 cells, were evaluated following 24 h incubation with serial dilutions of ring-substituted 1-hydroxynaphthalene-2-carboxanilide derivatives. The tested compounds differed between each other in the nature or in the position of substituents on the aromatic ring of the anilide moiety, whole structures of their molecules are shown in Table 1. The results of the WST-1 assay revealed that the derivatives with substituents introduced at *meta*- or *para*-positions exerted the cell proliferation inhibitory effect in both THP-1 and MCF-7 cell lines, as summarized in Table 1. By this analysis, compound **10** was confirmed as the derivative with the strongest antiproliferative activity against the THP-1 cell line (IC_50_ 2.55 µM). Concurrently, all *meta*- and *para*-substituted derivatives caused the inhibition of proliferation in MCF-7 cells with the IC_50_ values less than 10 µM. In our assays, another salicylanilide derivative, niclosamide, also showed the antiproliferative effect in both cell lines upon 24 h incubation (IC_50_ 2.14 µM and 5.38 µM in THP-1 and MCF-7 cells, respectively). In contrast, none of the *ortho*-substituted derivatives **1**, **4**, or **7** were able to affect cell proliferation of neither THP-1 nor MCF-7 cells at the tested concentrations.

Simultaneously, we assessed the cytotoxic effects of previously tested compounds using erythrosin B exclusion test. In general, among 10 tested 1-hydroxynaphthalene-2-carboxanilide derivatives, only molecules substituted by -CF_3_ group impaired viability of both tumor cell lines used upon 24 h treatment, while compounds containing -F or -Br substituents exerted no or very slight cytotoxic activity (Table 1, Supplementary Figures S1 and S2). Nevertheless, the strongest effect was observed on MCF-7 cells treated by compound **10** (LC_50_ of 8.78 µM).

### 2.2. Effects of 1-Hydroxynaphthalene-2-carboxanilides on Cell Cycle Progression

**Figure 1:**
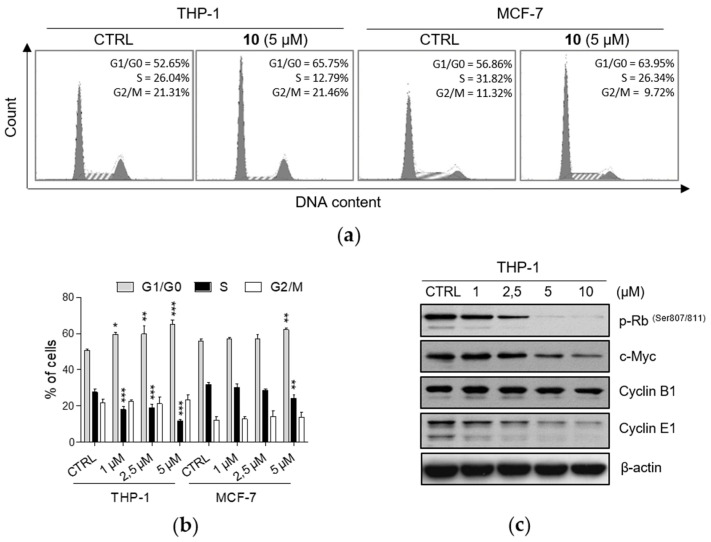
Compound **10** induces accumulation of THP-1 and MCF-7 cells in G1/G0 cell cycle phase. THP-1 or MCF-7 cells were treated with indicated concentrations of compound **10** for 24 h. (**a**) Representative histograms of DNA content in THP-1 and MCF-7 cells upon the treatment with compound **10**. Cell cycle distribution was determined using flow cytometry. (**b**) Results of distribution of THP-1 and MCF-7 cell in cell cycle phases are expressed as the mean ± SD from three independent experiments. * *p* < 0.05, ** *p* < 0.01, *** *p* < 0.001, significantly different from drug-free control (CTRL). (**c**) The levels of cell cycle regulators were detected by immunoblot analysis using appropriate antibodies. Representative immunoblots of one out of three experiments are shown. CTRL, drug-free control.

Based on the previous results demonstrating antiproliferative activity of *meta*- and *para*-substituted compounds, we have performed cell cycle analysis to investigate their effects on progression through cell cycle phases in THP-1 and MCF-7 cell lines. Our analysis revealed changes in cell cycle profiles upon 24 h treatment with compounds **2**, **3**, **5**, **6**, **8**, **9**, and **10**. For the experiments, we used the concentrations of compounds that significantly decreased cell proliferation without affecting cell viability or with a weak concurrent cytotoxic effect. In both cancer cell lines, the exposure of cells to tested compounds led to the accumulation of cells in G1/G0 cell cycle phase in a concentration-dependent manner together with the decrease of the percentage of cells in S phase, whereas the number of cells in G2/M phase was not altered (Figure 1a,b; Supplementary Figure S3). The changes in cell cycle distribution induced by tested compounds were qualitatively similar among all of them. On the other hand, the quantitatively strongest effect was observed upon the treatment by compound **10** in THP-1 cells. These results were additionally supported by the detection of decreased protein levels of cell cycle regulators controlling G1/S transition (Figure 1c). 24 h incubation with **10** resulted in reduced levels of the phosphorylated form of retinoblastoma protein [p-Rb (Ser 807/811)] and cyclin E1, both in a concentration-dependent manner. Moreover, levels of c-Myc were found to be down-regulated in the same manner. On the contrary, the levels of cyclin B1, the protein involved in G2/M progression, remained unchanged in our analyses.

### 2.3. Compound ***10*** Regulates STAT3 Signaling Pathway In Vitro

**Figure 2:**
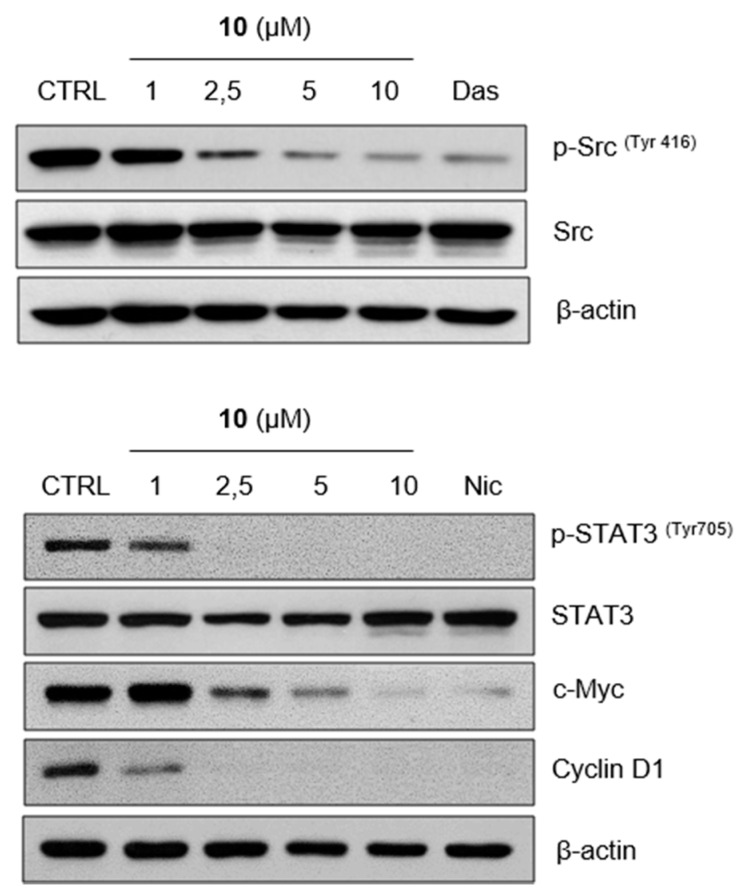
Compound **10** inhibits STAT3 signaling in breast cancer cells. MCF-7 cells were treated with compound **10** at indicated concentrations for 24 h. The levels of STAT3 and Src signaling pathway key players were detected by immunoblot analysis using appropriate antibodies. Samples treated by 20 µM of dasatinib (Das) or 10 µM of niclosamide (Nic) for 24 h were used as positive controls. Representative immunoblots of one out of three experiments are shown. CTRL, drug-free control.

As mentioned, niclosamide has been recently identified as the small molecule inhibitor of STAT3 [34,35], the member of a signaling pathway that regulates many cellular processes important for tumorigenesis, including cell proliferation, cell cycle progression, apoptosis, or tumor angiogenesis [39]. As demonstrated in Figure 2, compound **10** markedly reduced phosphorylation of STAT3 at tyrosine 705 in a concentration-dependent manner. Our results also showed inhibited phosphorylation of STAT3 induced by niclosamide, added as the positive control. Furthermore, we examined the effect of **10** on the phosphorylation of Src tyrosine kinase, the upstream tyrosine kinase of STAT3, and its potent direct activator [40]. We found dose-dependently decreased Src phosphorylation caused by **10** in 24 h treatment. That effect was comparable to the inhibition induced by dasatinib, potent Src kinase inhibitor [41]. Finally, the exposure to **10** resulted in the decrease of protein levels of c-Myc and cyclin D1 in a concentration-dependent manner as well.

### 2.4. Induction of Apoptosis by Compound ***10*** in THP-1 Cells

**Figure 3:**
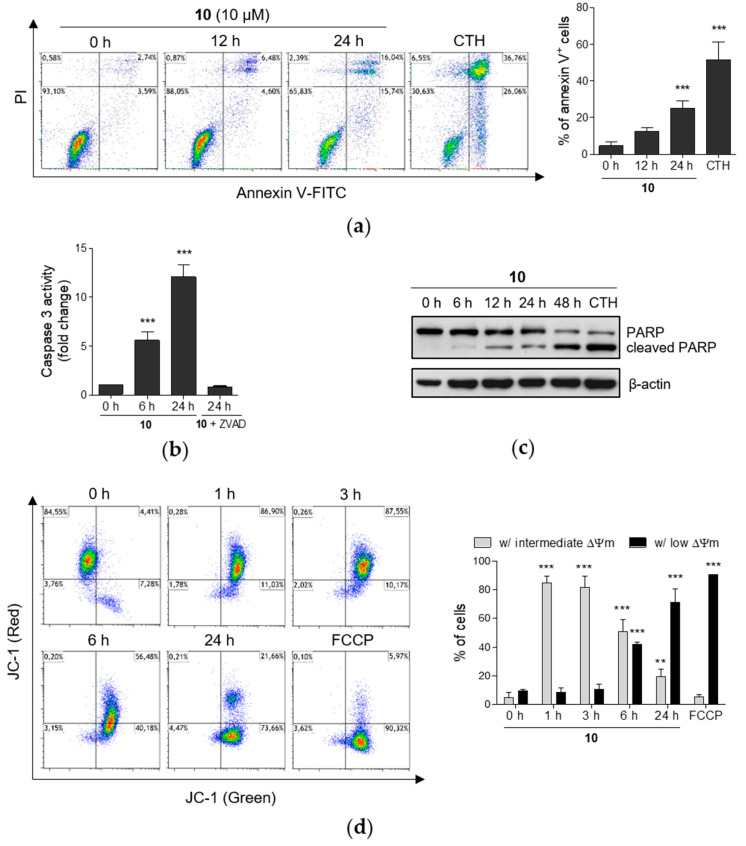
Compound **10** induces apoptosis in THP-1 cells. The same concentration of compound **10** (10 µM) was used for all experiments. (**a**) Cells were incubated with compound **10** for 12 h and 24 h, stained by annexin V-FITC conjugate and propidium iodide (PI) and analyzed by flow cytometry. Cells cultured with camptothecin (CTH, 5 µM) for 24 h were used as positive control. Representative flow cytometry plots are shown. (**b**) After the incubation of cells with compound **10**, cell lysates were prepared and incubated with caspase 3 substrate Z-DEVD-R 110 and the fluorescence was measured. Cells pretreated with pan caspase inhibitor Z-VAD-FMK (ZVAD) for 30 min before the incubation with **10** were used as negative control. (**c**) The levels of PARP and cleaved PARP were detected by immunoblot analysis. Sample treated by 5 µM of camptothecin (CTH) for 24 h was used as positive control. (**d**) THP-1 cells were stained with JC-1 fluorescent probe and the changes of the mitochondrial membrane potential (ΔΨm) were analyzed by flow cytometry. Cells treated by FCCP for 30 min were considered as positive control sample. Cell subsets with high red and green fluorescence were identified as the cells with intermediate MMP, cell subsets with low red, but high green fluorescence were determined as the cells with low MMP as shown in representative flow cytometry plots. The results are shown as the mean ± SD from at least three independent experiments. ** *p* < 0.01, *** *p* < 0.001, significantly different from 0 h.

Apart from the fact that the compound **10**, even at the lower concentration of 1 µM, induced a significant (*p* < 0.05) rise in the proportion of THP-1 cells in G1/G0 phase (Figure 1a,b), the higher concentrations starting from 10 µM impaired cell viability upon 24 h exposure (Supplementary Figure S1). Further, flow cytometric analysis showed significant (*p* < 0.001) increase in the percentage of annexin V-positive cells upon 24 h exposure to the same concentration of compound **10** (Figure 3a). Since those results indicated a time-dependent increase in the number of apoptotic cells, we decided to further explore the mechanism of cell death induced by **10**. We have assessed the activity of caspase 3, the effector caspase that is responsible for the cleavage of few pivotal cellular substrates, e.g., poly(ADP-ribose)polymerase (PARP) in the process of programmed cell death (Figure 3b,c) [42]. Compound **10** increased caspase 3 activity in a time-dependent manner. Moreover, the additional pretreatment by the pan-caspase inhibitor Z-VAD-FMK effectively suppressed such activity (Figure 3b).

Caspase 3 activation is preceded by a cascade of multiple cellular processes. Changes of mitochondrial membrane potential (MMP) can play an important role in the mitochondrial, or intrinsic, apoptotic pathway, and thus dissipation of mitochondrial membrane potential can be followed by cytochrome c release, formation of apoptosome activation of caspase 9, and final activation of caspase 3 [43]. To determine the impact of compound **10** on the alterations of MMP, we used JC-1 sensitive probe and flow cytometry. Within 1 h incubation, a decrease in MMP to intermediate level was detected (Figure 3d). Cell population with intermediate MMP has increased green fluorescence, but still maintain high red fluorescence. Thus, cells in that subset have most likely lost part of their MMP, but not all. Besides the cell subsets with high or low MMP, the third cell populations with intermediate MMP have been also identified in several models of apoptosis, e.g., in U937 cells treated by stavudine [44]. The subsequent loss of MMP in THP-1 cells was determined after longer incubation times with compound **10** up to 24 h treatment that led to the massive dissipation of MMP. Population of cells with low MMP under the treatment by **10** reached 72% in comparison to 91% of cells with low MMP after the exposure to FCCP, the uncoupler of mitochondrial oxidative phosphorylation used as positive control [45]. As mentioned earlier, the release of cytochrome c could follow the mitochondrial membrane depolarization and thus indicate the presence of the apoptotic process in cells [46]. Flow cytometric analysis showed a time-dependent increase in the number of the cells with released cytochrome c caused by compound **10** (Supplementary Figure S4).

### 2.5. Compound ***10*** Induces the Generation of Mitochondrial Superoxide in THP-1 Cells

**Figure 4:**
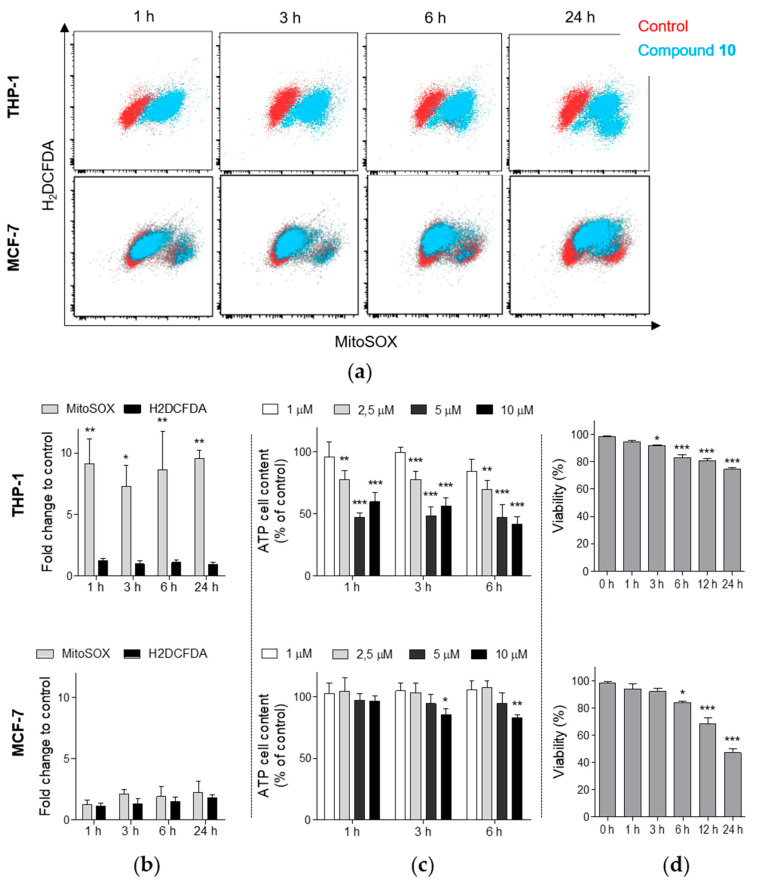
Compound **10** treatment results in loss of ATP cellular content and generation of mitochondrial superoxide in THP-1 cells. THP-1 and MCF-7 cells were treated with compound **10** (10 µM) if not stated otherwise for indicated time points. (**a**,**b**) THP-1 and MCF-7 cells were stained with MitoSOX Red or H_2_DCFDA and analyzed by flow cytometry. Dot plots from representative analysis are shown in panel A. (**c**) ATP cellular content was analyzed using luminescent assay. (**d**) Cell viability was determined by erythrosin B exclusion test at indicated time points. The results are shown as the mean ± SD from at least three independent experiments. * *p* < 0.05 ** *p* < 0.01, *** *p* < 0.001, significantly different from drug-free control (**b**,**c**) or 0 h (**d**).

Since mitochondrial membrane depolarization could be also related to the enhanced generation of RONS, in the next step, we examined the changes in production of diverse RONS using H_2_DCFDA and mitochondrial superoxide anion levels using MitoSOX fluorescent probe after the treatment with compound **10**. In THP-1 cells, a strong increase (nine-fold compared to control) in mitochondrial superoxide was observed even after 1 h incubation with 10 µM of compound **10** (Figure 4a,b). The elevated levels of superoxide anion were stable through the measured time points up to the 24 h incubation. In contrast, the analysis using H_2_DCFDA did not identify any significant increase in formation of the other RONS species in comparison with control. Moreover, additional analysis of total ATP cellular content showed a rapid time-dependent reduction in ATP levels in THP-1 (Figure 4c). For instance, compound **10** (10 µM) induced 40% and 59% reduction of ATP cellular content after 1 h and 6 h incubation, respectively. Our additional analyses also revealed a time-dependent cytotoxic effect of compound **10** in THP-1 cells (Figure 4d). Interestingly, a different effect was detected in MCF-7 cells. Although compound **10** induced time-dependent cytotoxic effect also in MCF-7 (Figure 4d), no associated significant generation of any of the RONS species was observed (Figure 4a,b) and we have determined a slighter effect on the reduction of ATP levels in MCF-7 cells compared to the effect in THP-1 (Figure 4c).

### 2.6. Compounds ***9*** and ***10*** Induces Inhibition of Proliferation in MCF-7 Xenografts

**Figure 5:**
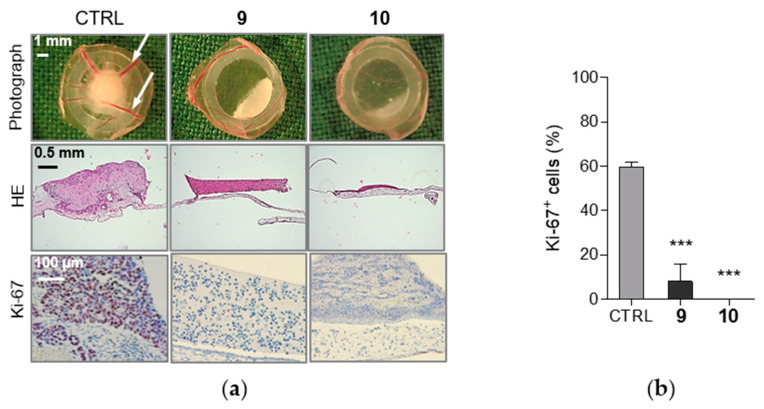
Compounds **9** and **10** inhibit proliferation of MCF-7 breast cancer xenografts. (**a**) MCF-7 breast cancer xenografts were topically treated with **9** or **10** (10 µM) for three days. 24 h later, xenografts were extracted (1st row), white arrows indicate tumor nourishing blood vessels. Paraffin-embedded MCF-7 xenografts were stained with hematoxylin and eosin (HE, 2nd row), original magnification 50×. The proliferation was analyzed by immunohistochemical staining of xenografts using proliferation marker, Ki-67 antigen (red nuclear stain, 3rd row). Representative microscopic pictures are shown, original magnification 200×. (**b**) The graph demonstrates the percentage of the Ki-67 positive, proliferating cells based on immunohistochemical staining of xenografts as described in (**a**). To calculate the proportion of marker positive and negative cells, 100–400 cells per tumor were evaluated. Data are mean ± SEM of *n* = 3 tumors/group. *** *p* < 0.001, significantly different from drug-free control (CTRL).

Following the initial study of cell proliferation inhibition, we decided to support the results obtained from in vitro assays by evaluation in a preclinical in vivo model. In oncology-related research, the chorioallantoic membrane (CAM) of fertilized chick eggs has been established for the investigation of, for example, tumor growth and tumor biology or the assessment of efficacy and toxicity of therapeutic compounds [47,48,49]. Therefore, we performed additional analysis of antiproliferative effects on pre-established MCF-7 xenografts on the chorioallantoic membrane of fertilized chicken eggs using an antibody against the human proliferation antigen Ki-67. The assay revealed that compounds **9** and **10**, which formerly showed antiproliferative effects in MCF-7 in vitro, also significantly decreased expression of the antigen Ki-67 and thus inhibited the proliferation of MCF-7 breast cancer xenografts (Figure 5a,b).

## 3. Discussion

In our present research, we have explored the potential anticancer properties of novel ring-substituted 1-hydroxynaphthalene-2-carboxanilides, newly designed and synthesized salicylanilide derivatives. Hydroxynaphthalene carboxamide derivatives including 1-hydroxynaphthalene-2-carboxanilides were previously shown to exert the antimycobacterial activity against various mycobacterial strains [2,3,4,5,6]. In our study, we intended to extend the knowledge of their other promising biological properties. In terms of their anticancer potency, we have focused mainly on the determination of antiproliferative activity in two different cancer cell lines, through the effect on cell cycle regulation and pro-apoptotic activity.

By this investigation, we have continued in our research of those biological effects of hydroxynaphthalene carboxamide derivatives. Recently, we have reported a potent antiproliferative activity against THP-1 and MCF-7 cells and pro-apoptotic effects in THP-1 cells induced by nitro-substituted hydroxynaphthalene carboxamide derivatives [37]. These promising results prompted us to investigate such action of new series of hydroxynaphthalene carboxamides with slightly modified structure. With respect to stated requirements for the potential biological activities of salicylanilides, we have chosen to test the derivatives substituted by electron-withdrawing groups. These are known to be required for the potential biological activities of salicylanilides [26,50,51]. In correspondence with our previous study [37], among tested compounds **1**–**10**, only those with the *meta*- and *para*- (but not the *ortho*-) substituted molecules exerted the significant antiproliferative effect (the most effective ones with single-digit micromolecular IC_50_ values) against THP-1 and MCF-7 cell lines. The same relationship between the position of the anilide substituent and the biological activity of hydroxynaphthalene carboxamides was observed in the studies of their antimycobacterial effects [2,6]. Authors have reported that *meta*- and *para*-substituted derivatives exerted higher antimycobacterial activity than *ortho*-substituted ones. Thus, they concluded that *meta*- or *para*-substitution in the anilide part of the molecule is essential for biological effect and the authors have also proposed the explanation of that relationship. The *ortho*-position of the anilide substituents, unlike the *meta*- or *para*-positions, could change the spatial orientation of anilide moiety towards the naphthalene part of the molecule and thus it could modify the biological effects [2,6]. The most effective molecule **10** in our study, having IC_50_ values in both cancer cell lines comparable to those reached by niclosamide, contained two –CF_3_ groups on the anilide part of the structure. Multiple studies showed the preparation of niclosamide’s derivatives by replacing the nitro group with another convenient substituent in order to improve its anticancer activity and also to overcome its physicochemical limitations. Consistent with our results, the introduction of another electron-withdrawing substituent –CF_3_ on the anilide part of the molecule was found to be favorable [52,53,54].

One of the fundamental features of cancer cells is the ability to sustain uncontrolled and abnormal cell growth [55]. That is the result of many alterations occurring in cancer cells as the changes in expression and activity of cell-cycle regulating proteins, the dysfunction of cell-cycle checkpoints, but also the deregulation of oncogene or tumor suppressor genes as another hallmark of tumor cells [56,57]. Thus, the targeting of cell cycle regulation represents one of the current approaches to anticancer therapy. Our results of cell cycle analysis revealed that all compounds exerted the inhibitory effect on G1/S transition in both cancer cell lines. The similar effect was also observed in our previous study with the nitro-derivatives [37]. Thus, we can assume that the effect on cell cycle progression is qualitatively similar among all tested compounds previously proved to inhibit cell proliferation regardless of substituent character. On the other hand, the intensity of this effect seemed to correlate with the intensity of cell proliferation inhibition, thus it is most likely influenced by both, the type of substituent and its position. To further support obtained data, we have chosen **10** as a model compound with the strongest effect on the accumulation of THP-1 cells in G1 phase. Rb protein is a tumor suppressor that plays a crucial role in the G1/S transition as it was found to be responsible for the progression through G1 phase and then also through G1 restriction point [58] since the phosphorylation of Rb protein led to the increase in transcriptional activity of E2F that promotes the expression of, e.g., cyclin E [59]. It was found that 24 h treatment by **10** significantly decreased the levels of phosphorylated Rb protein and also of cyclin E1, that can form an active complex with cyclin-dependent kinase 2 necessary to promote the G1/S progression [60]. These data were in correspondence with the dose-dependent reduction of c-Myc protein levels, the oncogene frequently deregulated in cancer cells. Besides many cell processes, c-Myc is regulating cell proliferation mainly through stimulating cell cycle progression by targeting genes that encode proteins involved in cell cycle control [61]. Additionally, **10** decreased protein levels of c-Myc and cyclin D1 in MCF-7 cells. Both, c-Myc and cyclin D1, that also promote cell proliferation via G1 phase regulation, represent downstream target genes of STAT3 and also Src signaling [62,63,64]. The Src or STAT3 pathways are the attractive targets for anticancer therapy, thus Src kinase inhibitors or direct STAT3 inhibitors were shown to constrain cell proliferation and induce apoptosis in cancer cells. Concerning clinically approved Src kinase inhibitors we can name dasatinib and bosutinib, actually dual protein tyrosine kinases Bcr-Abl and Src inhibitors [65]. Since our results demonstrated inhibited phosphorylation of STAT3 and interestingly decreased the activation of the upstream Src protein kinase that is responsible for STAT3 activation, these results could indicate targeting of these pathways by **10**.

As mentioned before, **10** impaired viability of THP-1 cells in a dose- and time-dependent manner. That was observed with the occurrence of apoptotic markers such as time-dependent membrane translocation of the phosphatidylserine upon treatment by **10** or cleavage of PARP and correspondingly caspase 3 activation. In general, salicylanilides are supposed to target mitochondria functions that can contribute to the induction of apoptosis in cancer cells. A number of studies also reported such an effect of induced mitochondrial dysfunction in different cancer cell types by leading salicylanilide derivatives with potent anticancer properties, niclosamide. Those impaired mitochondrial functions were linked to induced mitochondrial membrane depolarization, production of mitochondria-specific ROS or decreased ATP levels [22,24,25,66]. Accordingly, **10** induced loss of mitochondrial membrane potential in THP-1 cells that was followed by cytochrome c release, one of the pro-apoptotic factors released from mitochondria to cytosol. Interestingly, these effects were associated with a remarkable increase in the production of mitochondrial superoxide in THP-1 cells rather than other RONS species. Khanim et al. observed a similar selective generation of mitochondrial superoxide in multiple myeloma (MM) cells upon the treatment by niclosamide thus it was proposed that mitochondrial superoxide plays a critical role in cell death induction by niclosamide in MM cells [24]. Treatment by **10** resulted in overproduction of mitochondrial superoxide associated with rapid decrease of cellular ATP rather in THP-1 cells than in MCF-7 cells. Thus, we can presume that, unlike MCF-7 cells, the mitochondrial oxidative stress most likely contributes to cell death induction in THP-1 cells.

## 4. Materials and Methods

### 4.1. Tested Compounds and Reagents

Tested compounds ring-substituted 1-hydroxynaphthalene-2-carboxanilides **1**–**10** were synthesized and supplied by the Department of Chemical Drugs, Faculty of Pharmacy, University of Veterinary and Pharmaceutical Sciences Brno, Czech Republic. The synthesis procedure and detailed structural characterization of compounds **1**–**9** [N-(2-Fluorophenyl)-1-hydroxynaphthalene-2-carboxamide (1), N-(3-Fluorophenyl)-1-hydroxynaphthalene-2-carboxamide (2), N-(4-Fluorophenyl)-1-hydroxy-naphthalene-2-carboxamide (3), N-(2-Bromophenyl)-1-hydroxynaphthalene-2-carboxamide (4), N-(3-Bromophenyl)-1-hydroxynaphthalene-2-carboxamide (5), N-(4-Bromophenyl)-1-hydroxynaphthalene-2-carboxamide(6), 1-Hydroxy-N-(2-trifluoromethylphenyl)naphthalene-2-carboxamide (7), 1-Hydroxy-N-(3-trifluoromethylphenyl)naphthalene-2-carboxamide (8), 1-Hydroxy-N-(4-trifluoromethylphenyl)naphthalene-2-carboxamide (9)] [2] and N-[3,5-bis(trifluoromethyl)phenyl]-1-hydroxynaphthalene-2-carboxamide (10) [67] have been reported previously.

Niclosamide, dasatinib and roscovitine were purchased from Abcam (Cambridge, UK), camptothecin from Sigma Aldrich (St. Louis, MO, USA). All tested compounds were dissolved in dimethyl sulfoxide (DMSO) from Sigma Aldrich. Their fresh solutions were prepared prior to each experiment, while the final concentration of DMSO in the assays never exceeded 0.1% (*v/v*). RPMI 1640 and DMEM culture media, phosphate-buffered saline (PBS), foetal bovine serum (FBS) and antibiotics (penicillin and streptomycin) were purchased from HyClone Laboratories, Inc. (GE Healthcare, Logan, UT, USA).

### 4.2. Cell Culture

The human monocytic leukemia THP-1 cell line and the human breast adenocarcinoma MCF-7 cell line were purchased from European Collection of Cell Cultures (Salisbury, UK). THP-1 cells were cultured in RPMI 1640 medium containing 2 mM l-glutamine and MCF-7 cells were maintained in DMEM culture medium. Both types of culture media were supplemented with the antibiotic solution (100 U/mL of penicillin, 100 µg/mL of streptomycin) and 10% FBS. Cells were maintained in an incubator at 37 °C in a humidified atmosphere containing 5% CO_2_.

### 4.3. Analysis of Cell Proliferation and Viability

Cell proliferation and viability of THP-1 and MCF-7 cells were determined 24 h after the treatment with tested compounds in a broad concentration range using Cell Proliferation Reagent WST-1 (2-(4-iodophenyl)-3-(4-nitrophenyl)-5-(2,4-disulfophenyl)-2H-tetrazolium) (Roche Diagnostics, Mannheim, Germany) or erythrosin B exclusion test by counting with a hemocytometer, respectively, as it was previously described [37,68]. The IC_50_ and LC_50_ values were determined using the nonlinear regression four-parameter logistic model using GraphPad Prism 5.00 software (GraphPad Software, San Diego, CA, USA). MCF-7 xenografts were established by seeding 1 × 10^6^ cells in medium/Matrigel (1:1, *v/v*) onto the chick chorioallantoic membrane (CAM) of 8 days old embryos. For the next three days, xenografts were topically treated daily with 20 µL of PBS/0.1% DMSO with or without compounds **9** or **10** (10 µM). After another 24 h, the xenografts were collected, fixed, paraffin-embedded, and cut into 5-µm slices. The sections were stained either with hematoxylin and eosin or with antibodies against the human proliferation antigen Ki-67 (DakoCytomation, Glostrup, Denmark) and counterstained with hematoxylin.

### 4.4. Flow Cytometric Analysis of Cell Cycle Distribution and Apoptosis

Following 24 h incubation with indicated concentrations of tested compounds, the progression of THP-1 or MCF-7 cells through the cell cycle was analyzed after their fixation with ethanol and staining with propidium iodide, as described [37]. For annexin V-FITC/PI staining, THP-1 cells were treated with compound **10** (10 µM) or camptothecin (5 µM). At indicated time points, cells were harvested and stained using annexin V-FITC Early Apoptosis Detection Kit (Cell Signaling Technology, Boston, MA, USA) according to the manufacturer’s instructions. The analysis was performed by flow cytometer Cell Lab QuantaSC (Beckman Coulter, Brea, CA, USA) in FL-1 (annexin V-FITC) and in FL-2 (PI) channels (laser: 488 nm) followed by data evaluation using Kaluza Flow Cytometry Analysis Software 1.2 from Beckman Coulter. Single stained control samples for each fluorophore were used for setting compensation.

### 4.5. Quantification of ATP Cellular Content

THP-1 and MCF-7 cells were seeded into 96-well plates and exposed to 1, 2.5, 5, or 10 µM of compound **10**. At indicated time points of incubation, the analysis of cellular ATP content was assessed using CellTiter-Glo Luminescent Assay (Promega, Madison, WI, USA) according to the manufacturer’s protocol. The luminescence was measured using Infinite M1000 PRO microplate reader (TECAN, Männedorf, Switzerland).

### 4.6. Detection of Mitochondrial Membrane Potential and Reactive Species Levels

The levels of mitochondrial superoxide anions were determined by MitoSOX Red and the production of other diverse reactive oxygen and nitrogen species (RONS) such as hydroxyl radical or peroxynitrite was assessed by H_2_DCFDA (2′,7′-dichlorodihydrofluorescein diacetate) [69]. Both dyes were purchased from Molecular Probes, Inc. (Eugene, OR, USA), according to the manufacturer’s protocol. THP-1 or MCF-7 cells were incubated with compound **10** (10 µM) for indicated times. Then, 5 µM MitoSOX and 10 µM H_2_DCFDA were added to the samples for the final 30 min of incubation and the following analysis was carried out by flow cytometry. Results are presented as the ratio of the mean fluorescence intensity (MFI) of the samples and the control. JC-1 probe (Molecular Probes, Inc.) was used to assess changes in the mitochondrial membrane potential (MMP). Following treatment of THP-1 cells with compound **10**, cells were labeled in culture media with 10 µg/ml JC-1 for the last 20 min and analyzed by flow cytometry. Cells treated with 20 µM FCCP [carbonyl cyanide 4-(trifluoromethoxy)phenylhydrazone] for 30 min were used as a positive control. Results are shown as the percentage of cells with high red and green fluorescence of JC-1 aggregates and JC-1 monomers, respectively (cells with intermediate MMP), and the percentage of cells with low fluorescence of JC-1 aggregates and high fluorescence of JC-1 monomers (cells with low MMP).

The analyses were performed using FACSVerse (Becton Dickinson, Heidelberg, Germany) in FITC and PE channels (laser: 488 nm), or Cell Lab QuantaSC (Beckman Coulter) in FL-1 and FL-2 channels (laser: 488 nm), while the data were analyzed with FlowJo software (TreeStar Inc., Ashland, OR, USA) or Kaluza Flow Cytometry Analysis Software 1.2 (Beckman Coulter).

### 4.7. Cytochrome c Release Analysis

Assaying cytochrome c release was examined using flow cytometry according to the Waterhouse et al. [70]. Briefly, THP-1 cells incubated with 10 µM of compound **10** for 3 or 6 h were resuspended in plasma membrane permeabilization buffer (50 µg/mL digitonin, 80 mM KCl and 1 mM EDTA in PBS) and the lysates were centrifuged. The harvested pellet was fixed with paraformaldehyde and incubated in blocking buffer (3% (*w/v*) bovine serum albumin; 0.05% (*w/v*) saponin in PBS) for 1 h at room temperature. Then, the pellet was stained with anti-cytochrome c antibody (Alexa Fluor 647 mouse anti-cytochrome c, Becton Dickinson) overnight at 4 °C. The fluorescence levels were analyzed using flow cytometer FACSVerse in APC channel (laser: 633 nm).

### 4.8. Caspase 3 Activity Assay

THP-1 cells were treated with compound **10** (10 µM) and lysed in lysis buffer (10 mM Tris-HCl, pH 7.5; 100 mM NaCl; 4 mM EDTA; 1% NP-40) at indicated time points. Protein concentrations of samples were measured by BCA assay. An equal volume of cell lysate and reaction buffer (20 mM HEPES, 10% glycerol (*v/v*), 2 mM DTT) including Z-DEVD–R110 substrate (Molecular Probes, Inc.) were mixed and incubated for 1 h in the dark at room temperature. The measurement of fluorescence was performed using Infinite M1000 PRO microplate reader (TECAN). Cells pretreated with the pan-caspase inhibitor Z-VAD-FMK (50 µM, Molecular Probes, Inc.) were used as a negative control.

### 4.9. Western Blotting

Collected cells were lysed in radioimmunoprecipitation assay (RIPA) buffer supplemented with phenylmethanesulfonyl fluoride (PMSF, 1mM), both from Cell Signaling Technology (Danvers, MA, USA), and protease and phosphatase inhibitor cocktails (Roche Diagnostics, Mannheim, Germany). SDS-PAGE and Western blotting were performed according to the previously described protocol [37]. Staining of membranes with anti-β-actin antibody (Santa Cruz Biotechnology, Santa Cruz, CA, USA) was used to assess equal sample loading. Antibodies against cyclin E1 (Clone: HE12, Cat. #: sc-247) and p-Rb [Ser 807/811] (sc-16670-R) were purchased from Santa Cruz Biotechnology. Antibody against cyclin B1 (ab2949) was purchased from Abcam (Cambridge, UK), cyclin D1 (92G2, 2978), c-Myc (D84C12, 5605), PARP (9542), Src (36D10, 2109), p-Src [Tyr 416] (D49G4, 6943), STAT3 (79D7, 4904), and p-STAT3 [Tyr 705] (D3A7, 9145) were purchased from Cell Signaling Technology.

### 4.10. Statistical Analysis

All experimental data are presented as the mean ± standard deviation (SD). Statistical significance between values was assessed by one-way analysis of variance (ANOVA) paired with Dunnett’s post hoc test using GraphPad Prism 5.00 software (GraphPad Software, San Diego, CA, USA) at levels of * *p* < 0.05, ** *p* < 0.01 and *** *p* < 0.001.

## 5. Conclusions

In summary, our study provides evidence that new halogenated hydroxynaphthalene carboxamides inhibit the proliferation of THP-1 or MCF-7 cells most likely via preventing cells from progression through G1/S transition, where such an effect was found to be modulated by character and position of anilide substituent. Additionally, we could suggest that the most active compound impaired the cell viability through mitochondria-mediated apoptosis. The present study thus extended our previous report of such effects mediated by nitro-substituted hydroxynaphthalene carboxamides, and we can conclude that hydroxynaphthalene carboxamides structure represents a model moiety with promising anticancer properties.

## Figures and Tables

**Table 1:**
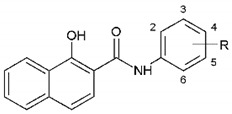
Antiproliferative and cytotoxic activities of ring-substituted 1-hydroxynaphthalene-2-carboxanilide derivatives. Cell proliferation and viability were determined using WST-1 analysis and erythrosin B exclusion test, respectively, after 24 h incubation with serial dilutions of tested compounds. Values shown are the mean ± SD from three independent experiments, each performed in triplicate.

Compound	R	THP-1		MCF-7	
		IC_50_ (μM)	LC_50_ (μM)	IC_50_ (μM)	LC_50_ (μM)
**1**	2-F	>20	>20	>20	>20
**2**	3-F	18.07 ± 2.71	>20	7.04 ± 1.28	>20
**3**	4-F	17.39 ± 1.76	>20	9.05 ± 0.16	>20
**4**	2-Br	>20	>20	>20	>20
**5**	3-Br	11.88 ± 2.27	>20	5.82 ± 1.30	>20
**6**	4-Br	9.39 ± 0.75	>20	7.09 ± 0.67	>20
**7**	2-CF_3_	>20	>20	>20	>20
**8**	3-CF_3_	9.20 ± 0.98	>20	4.59 ± 0.40	>20
**9**	4-CF_3_	9.16 ± 0.74	>20	3.82 ± 1.08	>20
**10**	3,5-CF_3_	2.55 ± 0.76	>20	3.80 ± 0.81	8.78 ± 0.58
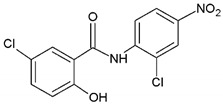					
Structure of niclosamide					
**Niclosamide**		2.14 ± 0.41	--	5.38 ± 0.90	--
**Roscovitine**		17.73 ± 1.55	--	>30	--
**Dasatinib**		38.94 ± 2.83	--	20.56 ± 0.87	--

## Supplementary Materials

Supplementary materials can be found at https://www.mdpi.com/1422-0067/21/10/3416/s1.

## Abbreviations

CAM	Chorioallantoic membrane
EGFR	Epidermal growth factor receptor
erbB	Erythroblastic oncogene B
FCCP	Carbonyl cyanide 4-(trifluoromethoxy)phenylhydrazone
IC50	Half maximal inhibitory concentration
LC50	Half maximal lethal concentration
MFI	Mean fluorescence intensity
MM	Multiple myeloma
MMP	Mitochondrial membrane potential
NF-κB	Nuclear factor kappa B
PARG	Poly (ADP-ribose) glycohydrolase
PARP	Poly (ADP-ribose) polymerase
PMSF	Phenylmethanesulfonyl fluoride
Rb	Retinoblastoma protein
RONS	Reactive oxygen and nitrogen species
STAT3	Signal transducer and activator of transcription 3
WST-1	2-(4-iodophenyl)-3-(4-nitrophenyl)-5-(2,4-disulfophenyl)-2H-tetrazolium
Z-DEVD-R110	(Z-ASP-GLU-VAL-ASP)2-rhodamine 110
Z-VAD-FMK	N-benzyloxycarbonyl-Val-Ala-Asp fluoromethyl ketone

## References

[1] Imramovsky A., Pauk K., Pejchal V., Hanusek J. (2011). Salicylanilides and Their Derivatives as Perspective Antituberculosis Drugs: Synthetic Routes and Biological Evaluations. Mini-Rev. Org. Chem..

[2] Gonec T., Kos J., Zadrazilova I., Pesko M., Keltosova S., Tengler J., Bobal P., Kollar P., Cizek A., Kralova K. (2013). Antimycobacterial and herbicidal activity of ring-substituted 1-hydroxynaphthalene-2-carboxanilides. Bioorg. Med. Chem..

[3] Gonec T., Pospisilova S., Kauerova T., Kos J., Dohanosova J., Oravec M., Kollar P., Coffey A., Liptaj T., Cizek A. (2016). N-Alkoxyphenylhydroxynaphthalenecarboxamides and Their Antimycobacterial Activity. Molecules.

[4] Kos J., Nevin E., Soral M., Kushkevych I., Gonec T., Bobal P., Kollar P., Coffey A., O’Mahony J., Liptaj T. (2015). Synthesis and antimycobacterial properties of ring-substituted 6-hydroxynaphthalene-2-carboxanilides. Bioorg. Med. Chem..

[5] Gonec T., Zadrazilova I., Nevin E., Kauerova T., Pesko M., Kos J., Oravec M., Kollar P., Coffey A., O’Mahony J. (2015). Synthesis and Biological Evaluation of N-Alkoxyphenyl-3-hydroxynaphthalene-2-carboxanilides. Molecules.

[6] Gonec T., Kos J., Zadrazilova I., Pesko M., Govender R., Keltosova S., Chambel B., Pereira D., Kollar P., Imramovsky A. (2013). Antibacterial and herbicidal activity of ring-substituted 2-hydroxynaphthalene-1-carboxanilides. Molecules.

[7] Macielag M.J., Demers J.P., Fraga-Spano S.A., Hlasta D.J., Johnson S.G., Kanojia R.M., Russell R.K., Sui Z., Weidner-Wells M.A., Werblood H. (1998). Substituted salicylanilides as inhibitors of two-component regulatory systems in bacteria. J. Med. Chem..

[8] Kauppi A.M., Nordfelth R., Hägglund U., Wolf-Watz H., Elofsson M. (2003). Salicylanilides are potent inhibitors of type III secretion in Yersinia. Adv. Exp. Med. Biol..

[9] Kos J., Zadrazilova I., Pesko M., Keltosova S., Tengler J., Gonec T., Bobal P., Kauerova T., Oravec M., Kollar P. (2013). Antibacterial and herbicidal activity of ring-substituted 3-hydroxynaphthalene-2-carboxanilides. Molecules.

[10] Zadrazilova I., Pospisilova S., Masarikova M., Imramovsky A., Ferriz J.M., Vinsova J., Cizek A., Jampilek J. (2015). Salicylanilide carbamates: Promising antibacterial agents with high in vitro activity against methicillin-resistant Staphylococcus aureus (MRSA). Eur. J. Pharm. Sci..

[11] Krátký M., Vinsová J. (2011). Salicylanilide ester prodrugs as potential antimicrobial agents—A review. Curr. Pharm. Des..

[12] Krátký M., Vinšová J., Buchta V. (2012). In vitro antibacterial and antifungal activity of salicylanilide pyrazine-2-carboxylates. Med. Chem..

[13] Baichwal R.S., Baxter R.M., Kandel S.I., Walker G.C. (1960). Antifungal action of salicylanilide. II. Can. J. Biochem. Physiol..

[14] Swan G.E. (1999). The pharmacology of halogenated salicylanilides and their anthelmintic use in animals. J. S. Afr. Vet. Assoc..

[15] Mudduluru G., Walther W., Kobelt D., Dahlmann M., Treese C., Assaraf Y.G., Stein U. (2016). Repositioning of drugs for intervention in tumor progression and metastasis: Old drugs for new targets. Drug Resist. Updat.

[16] Li Y., Li P.K., Roberts M.J., Arend R.C., Samant R.S., Buchsbaum D.J. (2014). Multi-targeted therapy of cancer by niclosamide: A new application for an old drug. Cancer Lett..

[17] Lu W., Lin C., Roberts M.J., Waud W.R., Piazza G.A., Li Y. (2011). Niclosamide suppresses cancer cell growth by inducing Wnt co-receptor LRP6 degradation and inhibiting the Wnt/beta-catenin pathway. PLoS ONE.

[18] Ye T., Xiong Y., Yan Y., Xia Y., Song X., Liu L., Li D., Wang N., Zhang L., Zhu Y. (2014). The anthelmintic drug niclosamide induces apoptosis, impairs metastasis and reduces immunosuppressive cells in breast cancer model. PLoS ONE.

[19] Wieland A., Trageser D., Gogolok S., Reinartz R., Höfer H., Keller M., Leinhaas A., Schelle R., Normann S., Klaas L. (2013). Anticancer effects of niclosamide in human glioblastoma. Clin. Cancer Res..

[20] Monin M.B., Krause P., Stelling R., Bocuk D., Niebert S., Klemm F., Pukrop T., Koenig S. (2016). The anthelmintic niclosamide inhibits colorectal cancer cell lines via modulation of the canonical and noncanonical Wnt signaling pathway. J. Surg. Res..

[21] Williamson R.L., Metcalf R.L. (1967). Salicylanilides: A new group of active uncouplers of oxidative phosphorylation. Science.

[22] Zhao J., He Q., Gong Z., Chen S., Cui L. (2016). Niclosamide suppresses renal cell carcinoma by inhibiting Wnt/β-catenin and inducing mitochondrial dysfunctions. Springerplus.

[23] Jin Y., Lu Z., Ding K., Li J., Du X., Chen C., Sun X., Wu Y., Zhou J., Pan J. (2010). Antineoplastic mechanisms of niclosamide in acute myelogenous leukemia stem cells: Inactivation of the NF-kappaB pathway and generation of reactive oxygen species. Cancer Res..

[24] Khanim F.L., Merrick B.A., Giles H.V., Jankute M., Jackson J.B., Giles L.J., Birtwistle J., Bunce C.M., Drayson M.T. (2011). Redeployment-based drug screening identifies the anti-helminthic niclosamide as anti-myeloma therapy that also reduces free light chain production. Blood Cancer J..

[25] Park S.J., Shin J.H., Kang H., Hwang J.J., Cho D.H. (2011). Niclosamide induces mitochondria fragmentation and promotes both apoptotic and autophagic cell death. BMB Rep..

[26] Liechti C., Sequin U., Bold G., Furet P., Meyer T., Traxler P. (2004). Salicylanilides as inhibitors of the protein tyrosine kinase epidermal growth factor receptor. Eur. J. Med. Chem..

[27] Zhang L., Hou L., Sun W., Yu Z., Wang J., Gao H., Yang G. (2016). Synthesis of p-O-Alkyl Salicylanilide Derivatives as Novel EGFR Inhibitors. Drug Dev. Res..

[28] Steffen J.D., Coyle D.L., Damodaran K., Beroza P., Jacobson M.K. (2011). Discovery and structure-activity relationships of modified salicylanilides as cell permeable inhibitors of poly(ADP-ribose) glycohydrolase (PARG). J. Med. Chem..

[29] Zhu X.F., Wang J.S., Cai L.L., Zeng Y.X., Yang D. (2006). SUCI02 inhibits the erbB-2 tyrosine kinase receptor signaling pathway and arrests the cell cycle in G1 phase in breast cancer cells. Cancer Sci..

[30] Fonseca B.D., Diering G.H., Bidinosti M.A., Dalal K., Alain T., Balgi A.D., Forestieri R., Nodwell M., Rajadurai C.V., Gunaratnam C. (2012). Structure-activity analysis of niclosamide reveals potential role for cytoplasmic pH in control of mammalian target of rapamycin complex 1 (mTORC1) signaling. J. Biol. Chem..

[31] Chen M., Wang J., Lu J., Bond M.C., Ren X.R., Lyerly H.K., Barak L.S., Chen W. (2009). The anti-helminthic niclosamide inhibits Wnt/Frizzled1 signaling. Biochemistry.

[32] Clevers H., Nusse R. (2012). Wnt/β-catenin signaling and disease. Cell.

[33] Dancey J. (2010). mTOR signaling and drug development in cancer. Nat. Rev. Clin. Oncol..

[34] Ren X., Duan L., He Q., Zhang Z., Zhou Y., Wu D., Pan J., Pei D., Ding K. (2010). Identification of Niclosamide as a New Small-Molecule Inhibitor of the STAT3 Signaling Pathway. ACS Med. Chem. Lett..

[35] Li R., You S., Hu Z., Chen Z.G., Sica G.L., Khuri F.R., Curran W.J., Shin D.M., Deng X. (2013). Inhibition of STAT3 by niclosamide synergizes with erlotinib against head and neck cancer. PLoS ONE.

[36] Musiol R. (2017). An overview of quinoline as a privileged scaffold in cancer drug discovery. Expert Opin. Drug Discov..

[37] Kauerova T., Kos J., Gonec T., Jampilek J., Kollar P. (2016). Antiproliferative and Pro-Apoptotic Effect of Novel Nitro-Substituted Hydroxynaphthanilides on Human Cancer Cell Lines. Int. J. Mol. Sci..

[38] Campos L.E., Garibotto F.M., Angelina E., Kos J., Tomašič T., Zidar N., Kikelj D., Gonec T., Marvanova P., Mokry P. (2019). Searching New Structural Scaffolds for BRAF Inhibitors. An Integrative Study using theoretical and experimental techniques. Bioorg. Chem..

[39] Al Zaid Siddiquee K., Turkson J. (2008). STAT3 as a target for inducing apoptosis in solid and hematological tumors. Cell Res..

[40] Yu H., Jove R. (2004). The STATs of cancer—New molecular targets come of age. Nat. Rev. Cancer.

[41] Roskoski R. (2015). Src protein-tyrosine kinase structure, mechanism, and small molecule inhibitors. Pharmacol. Res..

[42] Los M., Mozoluk M., Ferrari D., Stepczynska A., Stroh C., Renz A., Herceg Z., Wang Z.Q., Schulze-Osthoff K. (2002). Activation and caspase-mediated inhibition of PARP: A molecular switch between fibroblast necrosis and apoptosis in death receptor signaling. Mol. Biol. Cell.

[43] Waterhouse N.J., Goldstein J.C., von Ahsen O., Schuler M., Newmeyer D.D., Green D.R. (2001). Cytochrome c maintains mitochondrial transmembrane potential and ATP generation after outer mitochondrial membrane permeabilization during the apoptotic process. J. Cell Biol..

[44] Troiano L., Ferraresi R., Lugli E., Nemes E., Roat E., Nasi M., Pinti M., Cossarizza A. (2007). Multiparametric analysis of cells with different mitochondrial membrane potential during apoptosis by polychromatic flow cytometry. Nat. Protoc..

[45] Benz R., McLaughlin S. (1983). The molecular mechanism of action of the proton ionophore FCCP (carbonylcyanide p-trifluoromethoxyphenylhydrazone). Biophys. J..

[46] Gottlieb E., Armour S.M., Harris M.H., Thompson C.B. (2003). Mitochondrial membrane potential regulates matrix configuration and cytochrome c release during apoptosis. Cell Death Differ..

[47] Dagg C.P., Karnofsky D.A., Roddy J. (1956). Growth of transplantable human tumors in the chick embryo and hatched chick. Cancer Res..

[48] Nowak-Sliwinska P., Segura T., Iruela-Arispe M.L. (2014). The chicken chorioallantoic membrane model in biology, medicine and bioengineering. Angiogenesis.

[49] DeBord L.C., Pathak R.R., Villaneuva M., Liu H.C., Harrington D.A., Yu W., Lewis M.T., Sikora A.G. (2018). The chick chorioallantoic membrane (CAM) as a versatile patient-derived xenograft (PDX) platform for precision medicine and preclinical research. Am. J. Cancer Res..

[50] Guo L., Wang Q.L., Jiang Q.Q., Jiang Q.J., Jiang Y.B. (2007). Anion-triggered substituent-dependent conformational switching of salicylanilides. New hints for understanding the inhibitory mechanism of salicylanilides. J. Org. Chem..

[51] Waisser K., Bures O., Holý P., Kunes J., Oswald R., Jirásková L., Pour M., Klimesová V., Kubicová L., Kaustová J. (2003). Relationship between the structure and antimycobacterial activity of substituted salicylanilides. Arch. Pharm. (Weinheim).

[52] Mook R.A., Wang J., Ren X.R., Chen M., Spasojevic I., Barak L.S., Lyerly H.K., Chen W. (2015). Structure-activity studies of Wnt/β-catenin inhibition in the Niclosamide chemotype: Identification of derivatives with improved drug exposure. Bioorg. Med. Chem..

[53] Wu C.L., Chen C.L., Huang H.S., Yu D.S. (2018). A new niclosamide derivatives-B17 can inhibit urological cancers growth through apoptosis-related pathway. Cancer Med..

[54] Chen H., Yang Z., Ding C., Chu L., Zhang Y., Terry K., Liu H., Shen Q., Zhou J. (2013). Discovery of O-Alkylamino-Tethered Niclosamide Derivatives as Potent and Orally Bioavailable Anticancer Agents. ACS Med. Chem. Lett..

[55] Hanahan D., Weinberg R.A. (2011). Hallmarks of cancer: The next generation. Cell.

[56] Stewart Z.A., Westfall M.D., Pietenpol J.A. (2003). Cell-cycle dysregulation and anticancer therapy. Trends Pharmacol. Sci..

[57] Feitelson M.A., Arzumanyan A., Kulathinal R.J., Blain S.W., Holcombe R.F., Mahajna J., Marino M., Martinez-Chantar M.L., Nawroth R., Sanchez-Garcia I. (2015). Sustained proliferation in cancer: Mechanisms and novel therapeutic targets. Semin. Cancer Biol..

[58] Giacinti C., Giordano A. (2006). RB and cell cycle progression. Oncogene.

[59] Henley S.A., Dick F.A. (2012). The retinoblastoma family of proteins and their regulatory functions in the mammalian cell division cycle. Cell Division.

[60] Malumbres M., Barbacid M. (2009). Cell cycle, CDKs and cancer: A changing paradigm. Nat. Rev. Cancer.

[61] Bretones G., Delgado M.D., León J. (2015). Myc and cell cycle control. Biochim. Biophys. Acta.

[62] Leslie K., Lang C., Devgan G., Azare J., Berishaj M., Gerald W., Kim Y.B., Paz K., Darnell J.E., Albanese C. (2006). Cyclin D1 is transcriptionally regulated by and required for transformation by activated signal transducer and activator of transcription 3. Cancer Res..

[63] Barré B., Vigneron A., Coqueret O. (2005). The STAT3 transcription factor is a target for the Myc and riboblastoma proteins on the Cdc25A promoter. J. Biol. Chem..

[64] Haura E.B. (2006). SRC and STAT pathways. J. Thorac. Oncol..

[65] Wu P., Nielsen T.E., Clausen M.H. (2015). FDA-approved small-molecule kinase inhibitors. Trends Pharmacol. Sci..

[66] Zhou J., Jin B., Jin Y., Liu Y., Pan J. (2017). The antihelminthic drug niclosamide effectively inhibits the malignant phenotypes of uveal melanoma. Theranostics.

[67] Spaczynska E., Mrozek-Wilczkiewicz A., Malarz K., Kos J., Gonec T., Oravec M., Gawecki R., Bak A., Dohanosova J., Kapustikova I. (2019). Design and synthesis of anticancer 1-hydroxynaphthalene-2-carboxanilides with a p53 independent mechanism of action. Sci. Rep..

[68] Kollar P., Barta T., Zavalova V., Smejkal K., Hampl A. (2011). Geranylated flavanone tomentodiplacone B inhibits proliferation of human monocytic leukaemia (THP-1) cells. Br. J. Pharmacol..

[69] Setsukinai K., Urano Y., Kakinuma K., Majima H.J., Nagano T. (2003). Development of novel fluorescence probes that can reliably detect reactive oxygen species and distinguish specific species. J. Biol. Chem..

[70] Waterhouse N.J., Steel R., Kluck R., Trapani J.A. (2004). Assaying cytochrome C translocation during apoptosis. Methods Mol. Biol..

