# Supplementary material for: Ring-Substituted 1-Hydroxynaphthalene-2-Carboxanilides Inhibit Proliferation and Trigger Mitochondria-Mediated Apoptosis
Source: Int J Mol Sci. 2020 May 12;21(10):3416. doi: 10.3390/ijms21103416 (PMC7279329; doi:10.3390/ijms21103416)
Supplement: Supplementary file 1 [file ijms-21-03416-s001.pdf]

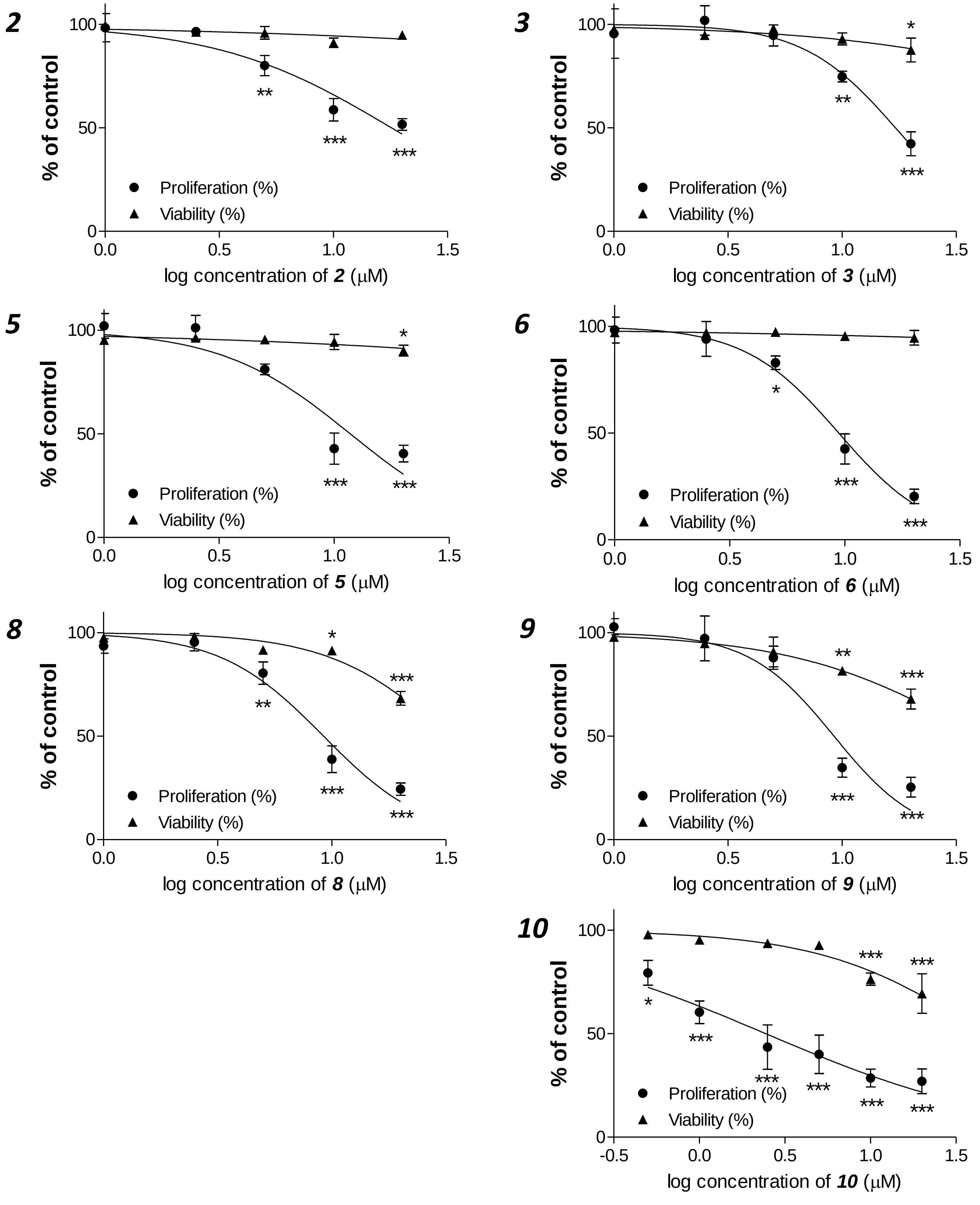

Figure S1. Antiproliferative and cytotoxic effects of 1-hydroxynaphthalene-2-carboxanilides in THP-1 cell line. Cells were cultured under the treatment by indicated concentrations of tested 1-hydroxynaphthalene-2-carboxanilide derivatives for 24h. Proliferation was determined using WST-1 assay, cell viability was assessed by erythrosin B exclusion test. The results are shown as the mean ± SD from at least three independent experiments. * $P < 0.05$ ** $P < 0.01$; *** $P < 0.001$, significantly different from drug-free control (CTRL).

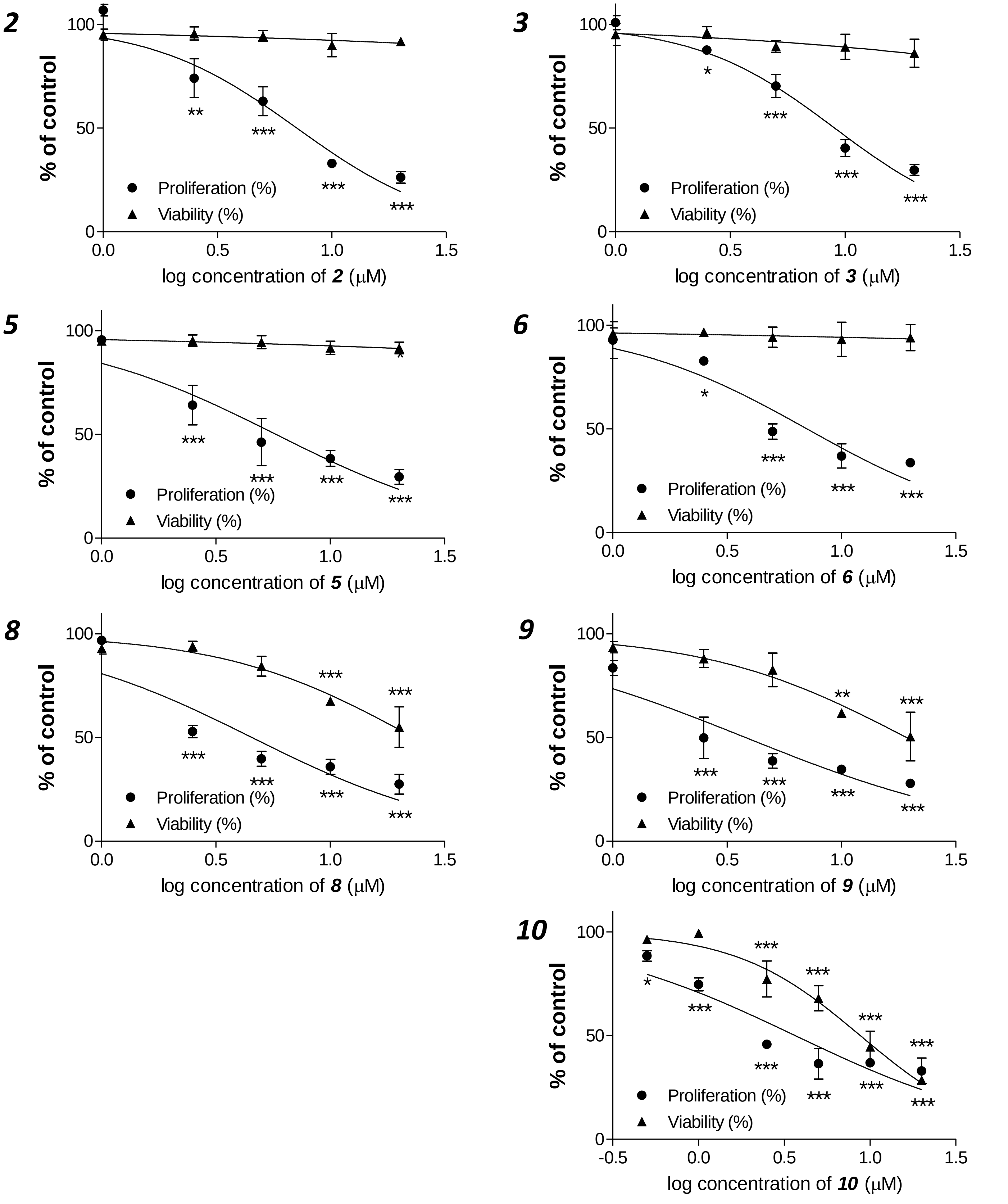

Figure S2. Antiproliferative and cytotoxic effects of 1-hydroxynaphthalene-2-carboxanilides in MCF-7 cell line. Cells were cultured under the treatment by indicated concentrations of tested 1-hydroxynaphthalene-2-carboxanilide derivatives for 24h. Proliferation was determined using WST-1 assay, cell viability was assessed by erythrosin B exclusion test. The results are shown as the mean ± SD from at least three independent experiments. * $P < 0.05$ ** $P < 0.01$; *** $P < 0.001$, significantly different from drug-free control (CTRL).

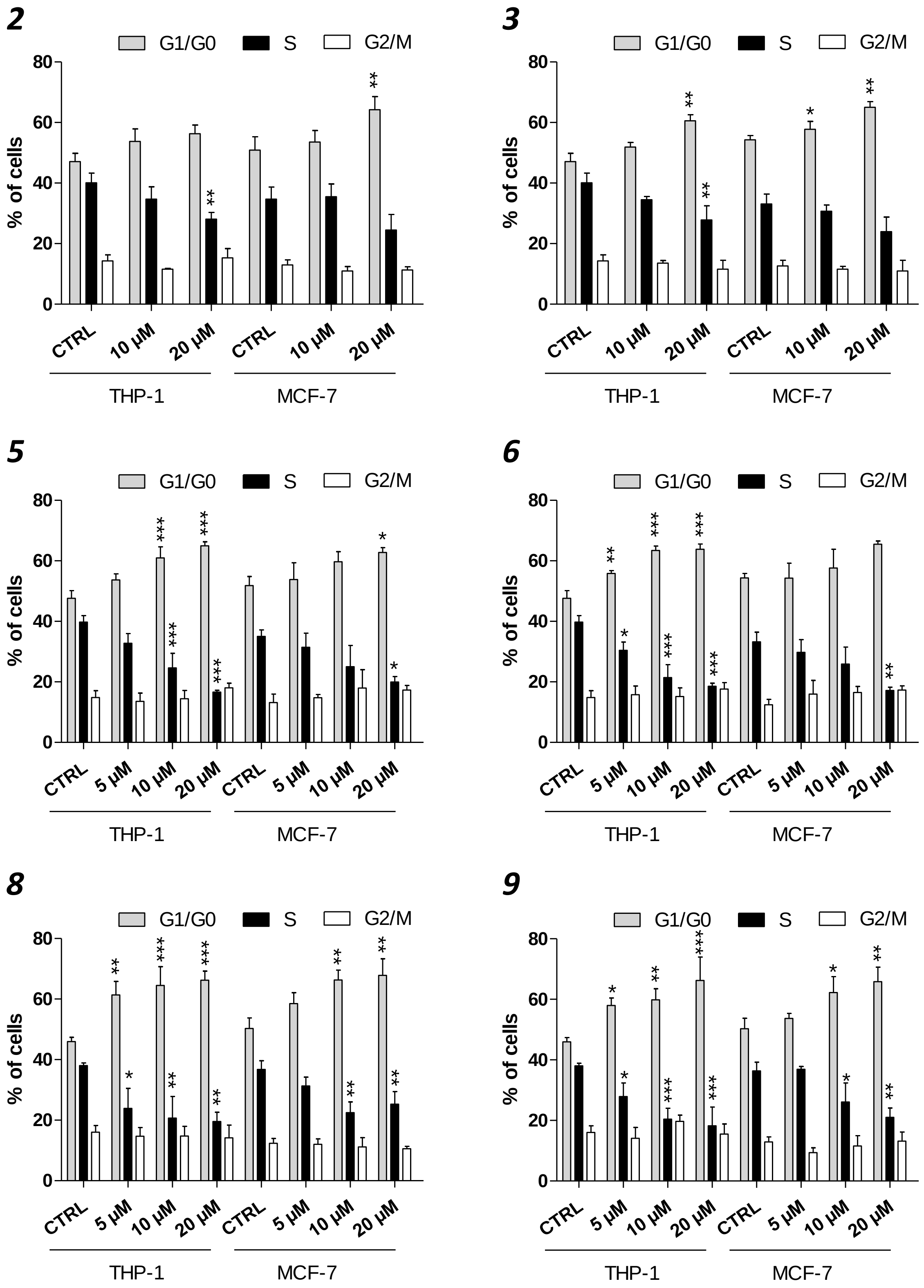

Figure S3. Treatment with 1-hydroxynaphthalene-2-carboxanilide derivatives induces dose-dependent accumulation of THP-1 and MCF-7 cells in G1/G0 cell cycle phase. Cell cycle distribution of THP-1 or MCF-7 cells upon the 24h treatment with indicated compounds. The results are expressed as the mean ± SD from three independent experiments. * $P < 0.05$; ** $P < 0.01$; *** $P < 0.001$, significantly different from drug-free control (CTRL).

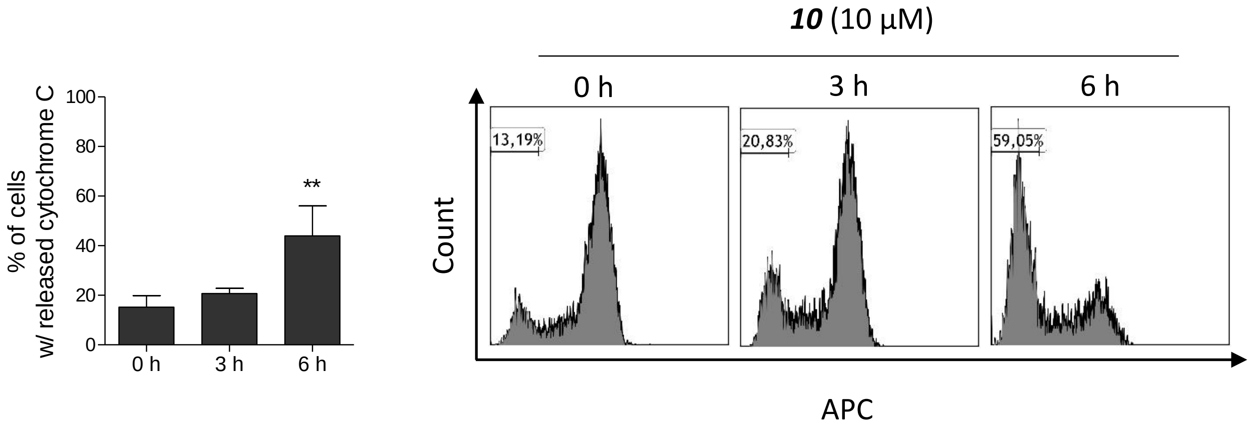

Figure S4. Treatment with 1-hydroxynaphthalene-2-carboxanilide derivative ***10*** induces cytochrome c release in THP-1 cells. THP-1 cells were treated with compound ***10*** and at indicated time points, cells were permeabilized and stained by cytochrome c antibody. The analysis was performed using flow cytometry in APC channel. The percentage of cells with low APC fluorescence were considered as the cells that had undergone cytochrome c release. The results are expressed as the mean ± SD from three independent experiments. ** $P < 0.01$, significantly different from 0 h.
